# Establishment of a value evaluation system for health and wellness tourism resources: Reflections on China’s official tourism resource evaluation criteria

**DOI:** 10.1371/journal.pone.0288749

**Published:** 2023-07-14

**Authors:** Meiqi Zhou, Huasong Luo, Juhua Hong, Dashuai Gao, Yukun Shen, Maolin Liang

**Affiliations:** 1 Faculty of Geography, Yunnan Normal University, Kunming, China; 2 Graduate School, Yunnan Normal University, Kunming, China; ESPOL Polytechnic University, ECUADOR

## Abstract

In recent years, China’s sub-healthy and aging populations have increased dramatically, giving rise to a series of health and wellness needs. People prefer health and wellness tourism activities instead of sightseeing tourism activities because of the increasing emphasis on safety and experience. Health and wellness tourism resources are a prerequisite for the development of health and wellness tourism. To critically reflect on China’s official tourism resource evaluation criteria, expert consultation was carried out by applying the Delphi method, and index weights were determined using the analytic hierarchy process. Through three rounds of expert consultation, a value evaluation system for health and wellness tourism resources was established and improved in three aspects: construction of an index system, selection of evaluation subjects, and grade discourse description, thus enhancing the feasibility and application value of the evaluation system. The system developed in this study not only enables a reflection on China’s official tourism resource evaluation criteria, but also presents a new approach in the value evaluation research of health and wellness tourism resources.

## Introduction

China is the most populous country in the world and is also home to the world’s largest elderly population. In the 19th century, population aging began to increase in China, raising the demand for health, medical care, and elderly care. However, there is a shortage of elderly care professionals, and the quantity and high-quality supply of medical facilities and elderly care service institutions are extremely mismatched with existing social demands [1]. Therefore, the overall demand is higher than the supply, and the social burden of elderly care is increasing. Simultaneously, an increase in fast-paced lifestyles, unhealthy eating habits, and food safety problems brought by urbanization endanger health, resulting in the proportion of sub-health groups increasing annually. Against the background of the serious subhealth and pension problems, in 2016 the China State Council published the *‘Health China 2030’ Planning Outline* which aimed to improve people’s physical and mental health, and to promote the initial development of the health industry. Accordingly, the China National Tourism Administration encouraged all regions to develop their health industry and actively develop health and wellness tourism resources. After the promulgation of China’s national standard named *National Health and Wellness Tourism Demonstration Base*, the construction of health and wellness tourism destinations was launched nationwide, promoting the overall development of health and wellness tourism in China.

*Huang Di Nei Jing*, the earliest Chinese medical book, recorded ancient people’s understanding and experience of health and wellness. Over time, the understanding of ‘health and wellness’ (*Kang Yang* in Chinese) has become multi-faceted. First, from the Chinese literal understanding, with ‘*Kang*’ for health and ‘*Yang*’ for nourishing of life, ‘*Kang Yang*’ means health and wellness. Secondly, from the perspective of industrial development, the word ‘*Yang*’ means pension, and ‘*Kang Yang*’ is defined as health and elderly care. In addition, China’s Office for Aging and *The Blue Book of Health and Wellness* are more inclined to define ‘*Kang Yang*’ as health, regimen, and elderly care. In *The Blue Book of Health and Wellness* published in 2017, health and wellness (*Kang Yang* in Chinese) is defined as activities that combine with the external environment to improve people’s body and mind, so that they can attain an optimal health condition. The concept of health and wellness tourism is clarified in the *National Health and Wellness Tourism Demonstration Base* (LB/T051-2016), which states that health and wellness tourism should include forms and functions such as longevity, healthy diet, and self-cultivation, with the aim of optimizing the physical and mental state of tourists [2]. Therefore, health and wellness tourism resources generally refer to tourism resources with health and wellness functions. For example, scholar Zhang Xi classifies health and wellness tourism resources as heavenly recreation, earthly recreation, water recreation, cultural recreation, and air-biology recreation [3]. *The Blue Book of Health and Wellness (2016)* subdivides health and wellness tourism resources into five types: forest, climate, ocean, hot spring, and Chinese medicine. According to the business patterns, Li Weijie divides health and wellness tourism resources into four categories: migratory bird type, healing type, rural type, and comprehensive type [4]. Thus, the classification of health and wellness tourism resources is not uniform, and it is more scientific to classify them according to local conditions and resource situations of research area.

At the end of the 19^th^ century, China began to explore how to scientifically survey existing tourism resources and prepared a trial draft of relevant census specifications. In 1997, the *Revised Plan of Tourism Resources Grading and Classification Program* was formulated, and a simple fuzzy quantitative evaluation method of expert scoring was designed. On this basis, the national standard of *Tourism Resources Classification*, *Survey and Evaluation* was first promulgated in 2003 [5]. The development of personalized tourism activities promoted the academic community’s re-understanding of tourism resources and the supply-side structural reform of tourism industry. After two revisions, a more complete *Classification*, *Investigation and Evaluation of Tourism Resources* (GB/T 18972–2017) was proposed in 2017 [6]. Tourism resources evaluation adopts the scoring evaluation method to quantitatively evaluate tourism resources, assigns points to each evaluation factor based on the importance of each element’s value, and divides the grade of tourism resources according to the total score of comprehensive evaluation. Therefore, the national standard for the classification, investigation, and evaluation of tourism resources provides the basis for research of tourism resource value evaluation. Scholars have discussed the rationality of China’s national standards for tourism resource evaluation [7, 8], and propose that based on national criteria, the overall value of representative tourism resources and different types of tourism resources in different places can be evaluated. However, China’s official evaluation criteria are universal, and the index system of value evaluation is slightly rough. To evaluate a certain type of tourism resources in a targeted way, it is necessary to build a more appropriate evaluation index system of tourism resources based on the official criteria and other value evaluation systems.

The sharp increase in the demand for health and old-age care ushered in health dividends, and the health and wellness tourism industry also ushered in a good development opportunity [9]. Health and wellness tourism is a novel form of tourism combining health, wellness, and elderly care functions. It caters to China’s current national conditions and diversified social needs, providing diversified health and wellness care functions and precise health care services to meet the needs of tourists for high-quality, multi-functional tourism. Compared developed countries that have dominant tourism markets [10], there is still a gap in China’s health and wellness tourism. More health and wellness tourism policies and goals began to appear in China in 2016, and a complete, large-scale and influential health and wellness tourism market has not yet been formed. Therefore, the three levels of government, academia and tourism should be integrated to adjust and upgrade the directions of policy and implementation, resource evaluation and development, and industry and culture integration [11]. The scientific evaluation of health and wellness tourism resources requires in-depth excavation of resource values, integration with related industries of the city as much as possible, echoing traditional culture, and enriching the function and value of health and wellness tourism resources [12]. Therefore, this study reconsiders relevant official national criteria in China; constructs a comprehensive evaluation system for the value of health and wellness tourism resources; and improves the evaluation model with three groups of experts, administrators, and tourists as the entry point. The establishment of a value evaluation system of health and wellness tourism resources is not only a reflection on China’s official evaluation criteria of tourism resources, but also a reference for the subsequent research of health and wellness tourism.

## Literature review

### Health and wellness tourism research

Existing research on health and wellness tourism on the Web of Science and CNKI databases is largely foreign and focuses on medical health and wellness tourism, hot spring health and wellness tourism, and rural health and wellness tourism. However, Chinese research focuses more on forest health tourism, the health and wellness industry, rural health and wellness tourism, and the construction of health and wellness tourism destinations. As an example of the latter, in the 19th century, forest bathing tours started to emerge in Germany. By the 20th century, the forest bathing base in Bad Wörishofen in Germany was regarded to be the earliest forest health and wellness tourism destination. As a natural oxygen bar, the forest is a tourism resource integrating rest, exercise, and wellness. It provides hiking, sunbathing, aerobic walking, and other special tourism projects [13]. Scholars have explored the spatial distribution of forest health and wellness tourism resources by means of geographic analysis methods such as nearest neighbor analysis and geographical concentration degree [14], thus laying the research foundation for forest health and wellness tourism development as well as the construction of tourism destinations.

From the perspective of demand and supply, Kaung Hwa proposed eight customer service elements of health and wellness tourism, including personnel service, environment, healthy diet, leisure, rehabilitation, unique travel experience, social activities, and physical and mental cultivation, and analyzed the health and wellness tourism preferences of elderly tourists [15]. Nikipelova et al. evaluated health and wellness tourism resources in Morsin and ranked it as a national resort. Health and wellness tourism has also become an emerging tourism industry in Pakistan [16]. Ullah et al. argued that the development of a new form of health and wellness tourism depends on the demand and supply of the tourist market and related industries, infrastructure, and external environments [17]. Taking Russia as an example, Dunets et al. concluded that social history, natural resources, economy, infrastructure, ethnic groups, tourism conditions, policies, and the Internet are components of the sustainable development of rural health and wellness tourism [18]. In rural health and wellness tourism activities, familiarization with traditional rural and farming cultures and experiencing the natural environment can meet the tourists’ different emotional needs and benefit physical and mental health.

The industrial revolution has promoted the rapid development of the healthcare industry, and modern medical technology has made the globalization of medical care possible. The problems of ethics and service quality supervision in the medical care system have hindered the rapid development of medical health and wellness tourism, which has thus attracted scholars’ attention and discussion. Karadayi-Usta and Serdar Asan propose a medical tourism service supply chain (MTSSC) model, which supplements the theoretical basis of medical health and wellness tourism service management [19]. The COVID-19 pandemic has brought numerous adverse negative impacts on the global tourism industry, and tourists are more likely to travel to countries or regions with better health care systems. Kaewkitipong et al. suggest that countries with abundant vaccines could develop medical health and wellness tourism to attract tourists from countries with a vaccine shortage to come for sightseeing and get vaccinated [20]. Rogerson and Rogerson found that South Africa is suitable for the development of health and wellness tourism as a climate resort that can treat diseases such as tuberculosis [21]. Ander Village in South Korea has utilized local herbal resources to develop health and wellness tourism and to realize economic recovery and growth [22]. Similarly, it is worth considering that traditional Chinese medicine is a precious resource with functions of medical care and health preservation that can serve the wellness tourism industry. Traditional Chinese Medicine (TCM) treatment has gradually highlighted its unique advantages in the practice of COVID-19 prevention and treatment. TCM tourism is a type of health and wellness tourism with Chinese characteristics. Wang believed that forest farms rich in TCM resources should deeply explore TCM cultural tourism resources on the basis of primary functions [23]. Guo analyzed the development status and problems of TCM tourism in Kaili City and put forward development suggestions from the perspective of consumers [24]. Ethnic medicine health care and longevity health tourism are ways to break through the development bottleneck of the pharmaceutical industry and realize the innovation and transformation of the tourism industry.

The relationship between supply and demand of health and wellness tourism, especially the demand side of tourists, such as psychological needs, physical needs, and the relationship between their motivations and behaviors, needs to be further discussed [25]. For example, in recent studies, Jiang et al. analyzed the diversified demands of health and wellness tourism from the perspective of intra-industry trade [9], and Liu et al. studied tourists’ motivation, inspiration and goal consistency [26]. Health and wellness tourism is highly service-oriented and subsequent research should be tourist-oriented and should focus on the construction of service functions of health and wellness tourism destinations.

### Tourism resource value evaluation research

The value evaluation research of tourism resources began in the middle of the 20th century, and the evaluation method was mainly qualitative. China’s tourism resource value evaluation started relatively late as compared to foreign research whereby tourism resource value evaluation research started in the 1970s. At the end of the 20th century, with the development of qualitative and quantitative evaluation methods, more subdivided research on the value evaluation of tourism resources appeared, and value evaluation of tourism resources showed a trend of refinement and diversification. Earlier qualitative evaluation can be traced back to the end of the 20th century, when Huang Minsheng assessed tourism resources in terms of resource value, development conditions, and benefits [27]. Most scholars used a three-level index system to construct the value evaluation system of tourism resources, such as resource value, abundance, tourism conditions, environment, and social economy, and then conducted empirical analysis with specific cases. Cases that have been studied include Shihezi in Xinjiang, Pianguan County, Lanzhou City, and Chongqing City. However, Wu (2008), and Chen and Wan (2021) constructed the evaluation index system from the aspects of brand value and environmental protection coefficients, practicality, hedonism, and symbolism of customer value experience [28, 29].

Based on tourism planning and the tourism value theory, Murtini et al. evaluated the tourism value of Ria Kenjeran beach from four aspects: attraction, infrastructure, suitability, and development strategy [30]. Velasco et al. evaluated the tourism value, socio-economic value, and market value of marine resources [31]. Belmonte Serrato et al. analyzed the scientific and educational value, accessibility, and tourism value of wasteland resources in Murcia [32]. Brzovska et al. used an experience economy model to evaluate the experience value of wine tourism [33], and Pantoja et al. evaluated suitability factors and resource advantages of beekeeping tourism in Chile [34]. However, Chekalina and Ryglová et al. established a multidimensional model for the strategic evaluation of tourism resources [35, 36], Hou and Li et al. innovatively introduced sensor technology and artificial intelligence neural network model to build tourism resource recommendation and evaluation model [37, 38]. Therefore, value evaluation of tourism resources has experienced a range of qualitative to quantitative evaluation methods, and from single to diversified evaluation contents. At present, different types of scientific evaluation systems have been formed in the academic community, which occupy an important position in tourism research. These value evaluation theories and methods of tourism resources have been studied in many fields, but research in the field of health and wellness tourism remains limited. Very few scholars have constructed the evaluation index system of forest health and wellness tourism and hot spring tourism from different perspectives, and there are few studies on the overall value evaluation index system of health and wellness tourism.

## Materials and methods

### Research methods

#### Delphi method

The Delphi method was created by RAND Corporation in 1946 and is widely used in comprehensive evaluation and scientific prediction studies in various disciplines [39]. This method constitutes several rounds of opinion consultations involving expert group members by means of questionnaires, and judgments and results are obtained when expert opinions converge. The number of panelists ranges between 15 to 40, and the stability of panelists is the key to using the Delphi method. It is a classic method of comprehensive evaluation research, which is suitable for the construction of the value evaluation index system of health and wellness tourism resources [40].

#### Analytic hierarchy process

In the late 20th century, American scholar T. L. Satty proposed the analytic hierarchy process (AHP) [41]. AHP combines qualitative and quantitative analysis to determine the weight of hierarchy comprehensively. Its application guideline is to divide a scientific problem into several levels, with each level being composed of several influencing factors. Then, the weight of each level of factors is assigned according to the degree of influence, and finally the combined weight of each level of indicators is obtained. Therefore, AHP simplifies complex scientific problems by splitting them into several levels. Comprehensive decision-making of AHP is widely applicable and can be applied to the weight determination of the value evaluation index system [42].

#### Fishbein-Rosenberg evaluation model

Rosenberg and Fishbein put forward the model of behavior and attitude measurement and evaluation in the same basic form [43]. This evaluation model is most often used when analyzing consumer decisions and in the comprehensive evaluation of tourist destinations. In the field of tourism resource evaluation, the Fishbein-Rosenberg evaluation model has a high application value and is a classic evaluation method. The calculation formula of the model is as follows.


$$E=\sum_{i=1}^{n}Q_{i}P_{i}$$

Where E is the comprehensive evaluation value, n is the total number of factors, Q_i_ is the weight of the i^th^ factor, and P_i_ is the evaluation score of the i^th^ factor. This study tries to improve the evaluation model by introducing the evaluation opinions of experts, administrators, and tourists, and assigning different weights to make it more suitable for the value evaluation of health and wellness tourism resources.

### Primary selection of indicators

The traditional three-level system is adopted to construct the evaluation index system of health and wellness tourism resources. The selection of the first-level index is mainly based on the first-level categories in *Classification*, *Investigation and Evaluation of Tourism Resources* (GB/T 18972–2017), that is, resource elements’ value, environmental value, and development and construction value are selected as the three first-level indicators. When selecting the second-level index, on the basis of China’s relevant evaluation criteria and domestic and foreign studies, the index is modified and added according to the characteristics of health and wellness tourism resources. Nine second-level indexes are selected, including tourism health value, cultural value, resource dependency, resource added value, environment quality, diversity, stability, location characteristics, and construction conditions. In the selection of the three-level index, starting from the three main characteristics of health, regimen, and elderly care, and referring to the index system constructed by domestic and foreign scholars, a total of 36 indicators are determined.

### Data type, source, and method of data collection

Adhering to the principle of objectivity and comprehensive evaluation, the information of experts studying health and wellness tourism was randomly searched on the literature platform. Scholars were invited to participate in the expert consultation by e-mail and telephone, and 35 interviewed experts who voluntarily participated in the whole consultation were identified. The interviewed expert group was composed of experts in tourism geography, ecology, Chinese Traditional Medicine and other research fields, among which 28 were engaged in tourism research, accounting for 80%. This study was approved by *the Biomedical Research Ethics Committee*. Participant experts provided consent via email and telephone communication, thus the experts involved throughout the consultation process voluntarily agreed to participate in the study.

The expert consultation questionnaire included three parts: the judgment of the importance of primary indexes, rationality of index system, and suggestions of index correction. Based on the increasing order of importance of indexes, they were divided into five dimensions, and assigned as 1, 3, 5, 7 and 9 points in turn. The first round of expert consultation was conducted from August 17, 2021, to August 29, 2021. A total of 35 expert consultation forms were sent out by email and all of them were collected. After analyzing the first round of expert consultation, the index system was modified, and a second round of questionnaires was formed. The second round of expert consultation took place from December 1, 2021, to December 8, 2021, with no change in the list of experts. A total of 35 expert consultation forms were distributed and all of them were collected. All the consultation questionnaires were completed and valid, and the effective recovery rates of the first and second rounds were 100%.

The final index system was determined through the first two rounds of expert consultation, and the pairwise importance comparison matrix of three levels was constructed respectively to form the relative importance assignment matrix table. The relative importance of the pairwise index was compared by using the 1−9 fractional scale method, and the third round of expert consultation was continued. Experts participating in the first two rounds were invited to fill in the relative importance assignment matrix table between January 5, 2022, and January 12, 2022. A total of 35 consultation questionnaires were sent out and all of them were collected. All the consultation questionnaires were completed and valid. The effective recovery rate of the third round of expert consultation questionnaires was 100%.

### Degree of concentration, dispersion, and coordination

Concentration degree (M) can reflect the importance of indicators. The larger the concentration degree, the more important the indicators that need to be retained. Usually, if M is between 7 and 9, the corresponding indicators can be retained. If M is between 5 and 7, the two indexes of dispersion and coordination should be combined, or the choice should be made according to experts’ opinions and characteristics of health and wellness tourism resources. If M is less than 5, it is recommended to delete the indicator directly. Dispersion degree (S) is used to describe the dispersion of the evaluation of various indicators by experts. Generally, the second round of expert consultation can only be entered when S is higher than 0.63. Coordination degree (V) is used to measure the coordination of experts’ opinions. When V is between 0 and 0.33, the coordination degree is high, and the opinions among experts are more unified, so relevant indicators can be retained. The calculation formulas of the concentration, dispersion, and coordination degrees are as follows.


$$M=\frac{1}{n}\sum_{k=1}^{n}Q_{k}$$

$$S=\sqrt{\frac{1}{n}\sum_{k=1}^{n}{\left(Q_{k}-M\right)}^{2}}$$

$$V=\frac{S}{M}$$

Where n is the total number of experts interviewed, k is the expert serial number, and Q_k_ is the value of the kth expert on the indicator.

## Results

### Modified evaluation index system

#### First round of expert consultation

In the results of the first round of expert consultation, the mean concentration degree of evaluation indicators was 6.82, the maximum was 8.26, and the minimum was 5.23. The mean dispersion degree was 1.57, the maximum was 2.08, and the minimum was 0.99. The mean coordination degree was 0.24, the maximum was 0.35, and the minimum was 0.12 (Table 1). In the first level of resource elements’ value, coordination degrees of cultural value of filial piety, resource integrity, and resource awareness were greater than or equal to 0.33, indicating that the importance and coordination of these three indicators are weak. Therefore, the filial piety cultural value, resource integrity, and resource awareness were deleted. Accordingly, based on the revised suggestions put forward by experts, the two second-level indexes of resource dependency and resource added value were merged into resource dependency and added value, and diversity and stability were merged into one index of diversity and stability. In the second level, diversity of species and stability of ecological environment were sufficient to support diversity and stability, so the two indexes were deleted. On the layer of development and construction value, experts suggested that tourism facilities and infrastructure should be merged. Social and economic conditions can better express the level of economic development, and land use conditions and environment capacity have been reflected in other indicators. Therefore, the three indicators of land use conditions, tourism facilities, and environment capacity were deleted, and the expression of economic development level was replaced with socio-economic conditions. In addition, based on the current situation of public health emergencies, two indicators of medical and health services and public health incident plan were added.

**Table 1:** Results of the first round of expert consultation.

First level	Second level	Third level	M	S	V
Resource elements’ value	Tourism health value	Health preservation value	8.26	1.08	0.13
		Ornamental recreation value	6.26	1.66	0.27
		Scientific elderly care value	7.29	1.86	0.26
		Rehabilitation and convalescence value	7.34	1.76	0.24
	Cultural value	Cultural value of filial piety	5.23	1.84	0.35
		Cultural value of TCM physiotherapy	6.66	1.55	0.23
		Cultural value of health preservation	6.89	1.65	0.24
		Cultural value of scientifical education	5.69	1.75	0.30
	Resource dependency	Resource integrity	6.09	1.99	0.33
		Resource scale and abundance	6.66	1.82	0.27
		Specificity of resources	6.89	1.72	0.25
		Combination degree of resources	6.77	1.84	0.27
	Resource added value	Resource impact	6.03	1.75	0.29
		Resource awareness	6.31	2.08	0.33
Environmental value	Environment quality	Temperature	7.63	1.24	0.16
		Humidity	6.83	1.46	0.21
		Altitude	6.37	1.84	0.29
		Forest coverage rate	7.29	1.67	0.23
		Air quality index	8.20	1.19	0.15
		Noise	7.69	1.35	0.18
		Centralized treatment rate of domestic sewage	6.89	1.43	0.21
		Harmless disposal rate of household garbage	7.06	1.47	0.21
	Diversity	Diversity of ecological environment	6.89	1.72	0.25
		Diversity of species	6.37	1.64	0.26
	Stability	Stability of ecological environment	6.60	1.57	0.24
		Stability of species	5.97	1.61	0.27
Development and construction value	Location characteristics	External traffic conditions	8.09	0.99	0.12
		Distance from main source of visitors	6.48	1.87	0.29
		Similarities and differences with nearby tourist destinations	6.08	1.46	0.24
		Distance from nearby tourist destinations	5.74	1.44	0.25
	Construction conditions	Infrastructure	8.26	1.08	0.13
		Land use conditions	6.20	1.67	0.27
		Economic development level	6.54	1.44	0.22
		Tourist facilities	7.69	1.17	0.15
		Environment capacity	7.11	1.43	0.20
		Policies and regulations	7.06	1.62	0.23

Note: TCM: traditional Chinese medicine.

#### Second round of expert consultation

In the results of second round of expert consultation (Table 2), the mean concentration degree of evaluation indicators was 7.15, the maximum was 8.49, and the minimum was 5.74. The mean dispersion degree was 1.59, the maximum was 2.24, and the minimum was 0.89. The mean coordination degree was 0.23, the maximum was 0.38, and the minimum was 0.10.

**Table 2:** Results of the second round of expert consultation.

First level	Second level	Third level	M	S	V
Resource elements’ value	Tourism health value	Health preservation value	8.20	1.11	0.13
		Ornamental recreation value	6.83	1.84	0.27
		Scientific elderly care value	7.51	1.77	0.24
		Rehabilitation and convalescence value	7.86	1.63	0.21
	Cultural value	Cultural value of TCM physiotherapy	6.43	1.65	0.26
		Cultural value of health preservation	7.11	1.60	0.23
		Cultural value of scientifical education	6.09	1.90	0.31
	Resource dependency and added value	Resource scale and abundance	6.49	1.96	0.30
		Specificity of resources	6.60	1.87	0.28
		Resource impact	6.09	1.90	0.31
		Combination degree of resources	6.03	2.24	0.37
Environmental value	Environment quality	Temperature	8.09	1.12	0.14
		Humidity	7.57	1.42	0.19
		Altitude	7.17	1.48	0.21
		Forest coverage rate	7.69	1.37	0.18
		Air quality index	8.20	1.21	0.15
		Noise	8.09	1.40	0.17
		Centralized treatment rate of domestic sewage	7.29	1.82	0.25
		Harmless disposal rate of household garbage	7.23	1.80	0.25
	Diversity and stability	Diversity of species	6.31	1.60	0.25
		Stability of ecological environment	6.60	1.67	0.25
Development and construction value	Location characteristics	External traffic conditions	8.14	1.55	0.19
		Distance from main source of visitors	7.00	1.68	0.24
		Similarities and differences with nearby tourist destinations	6.20	1.76	0.28
		Distance from nearby tourist destinations	5.74	2.17	0.38
	Construction conditions	Medical and health services	8.49	0.89	0.10
		Infrastructure	8.31	1.18	0.14
		Public health incident plan	7.63	1.44	0.19
		Policies and regulations	7.17	1.64	0.23
		Socio-economic conditions	6.43	1.14	0.18

Note: TCM: traditional Chinese medicine.

Coordination degrees of ‘combination degree of resources’ and ‘distance from main source of visitors’ were greater than 0.33, and the importance and coordination of these two indicators were lacking, therefore these were deleted. The data analysis results of the other indicators were good and retained. After the second round of expert consultation, a revised and confirmed value evaluation system of health and wellness tourism resources was constructed (Table 3).

**Table 3:** Final evaluation index system of health and wellness tourism resources.

General objective level	First level	Second level	Third level
Value of health and wellness tourism	Resource elements’ value	Tourism health value	Health preservation value
			Ornamental recreation value
			Scientific elderly care value
			Rehabilitation and convalescence value
		Cultural value	Cultural value of TCM physiotherapy
			Cultural value of health preservation
			Cultural value of scientifical education
		Resource dependency and added value	Resource scale and abundance
			Specificity of resources
			Resource impact
	Environmental value	Environment quality	Temperature
			Humidity
			Altitude
			Forest coverage rate
			Air quality index
			Noise
			Centralized treatment rate of domestic sewage
			Harmless disposal rate of household garbage
		Diversity and stability	Diversity of species
			Stability of ecological environment
	Development and construction value	Location characteristics	External traffic conditions
			Distance from main source of visitors
			Similarities and differences with nearby tourist destinations
		Construction conditions	Medical and health services
			Infrastructure
			Public health incident plan
			Policies and regulations
			Socio-economic conditions

Note: TCM: traditional Chinese medicine.

### Evaluation model

After a consistency test, the consistency ratio (CR) of levels 1−3 was less than 0.1, therefore the model passed the consistency test. Then, index weights of each level were calculated (Table 4). In the first level, the weight difference of resource elements’ value, environmental value, and development and construction value were small, and the importance of resource elements’ value was slightly higher than that of environmental value and development and construction value. In the second level, construction conditions, environment quality, and tourism health value were the three most important dimensions. Resource dependency and added value, and cultural value occupied a lower proportion and were in a secondary position. The overall index system weights were established and can be applied to the empirical analysis of specific cases.

**Table 4:** Weights of the value evaluation index of health and wellness tourism resources.

General objective level	First level	Weight	CR	Second level	Weight	CR	Third level	Weight	CR
Value of health and wellness tourism	Resource elements’ value	0.3700	0.0000	Tourism health value	0.5395	0.0000	Health preservation value	0.2756	0.0007
							Ornamental recreation value	0.1232	
							Scientific elderly care value	0.2476	
							Rehabilitation and convalescence value	0.3537	
				Cultural value	0.2531		Cultural value of TCM physiotherapy	0.3335	0.0000
							Cultural value of health preservation	0.4436	
							Cultural value of scientifical education	0.2229	
				Resource dependency and added value	0.2074		Resource scale and abundance	0.3088	0.0051
							Specificity of resources	0.3439	
							Resource impact	0.3473	
	Environmental value	0.3329		Environment quality	0.5989	0.0000	Temperature	0.2306	0.0046
							Humidity	0.0720	
							Altitude	0.0645	
							Forest coverage rate	0.1047	
							Air quality index	0.1901	
							Noise	0.1160	
							Centralized treatment rate of domestic sewage	0.1053	
							Harmless disposal rate of household garbage	0.1166	
				Diversity and stability	0.4011		Diversity of species	0.3119	0.0000
							Stability of ecological environment	0.6881	
	Development and construction value	0.2970		Location characteristics	0.3809	0.0000	External traffic conditions	0.4239	0.0021
							Distance from main source of visitors	0.2507	
							Similarities and differences with nearby tourist destinations	0.3255	
				Construction conditions	0.6191		Medical and health services	0.2305	0.0047
							Infrastructure	0.2449	
							Public health incident plan	0.1857	
							Policies and regulations	0.1293	
							Socio-economic conditions	0.2095	

Note: TCM: traditional Chinese medicine.

Because health and wellness tourism activities involved related research experts, administrators, and tourists, it is segmentary to evaluate a place’s health and wellness tourism resources only from one perspective. Based on the above reasons, the Fishbein-Rosenberg evaluation model is innovatively improved when the evaluation opinions of experts, administrators, and tourists are given different weights. Thus, the value evaluation model of health and wellness tourism resources is established. The improved model is as follows.


$$E=\sum_{i=1}^{m}P_{i}Q_{i}*40\%+\sum_{i=1}^{n}P_{i}Q_{i}*30\%+\sum_{i=1}^{s}P_{i}Q_{i}*30\%$$

Where E is the total evaluation score; P_i_ is the evaluation score of respondents; Q_i_ is the weight value of evaluation factors; and m, n, and s represent the total number of surveyed experts, administrators, and tourists respectively.

In tourism activities, administrators represent the supply side and tourists represent the demand side. Both supply and demand are equal, so administrators and tourists are given the same weight. However, experts who study health and wellness tourism are often able to evaluate from a more scientific and comprehensive perspective, so the evaluation weight of experts is slightly higher than that of administrators and tourists in this model. Finally, the weights of this model are determined: the weights of experts, administrators, and tourists are 40%, 30%, and 30%, respectively.

### Grade of evaluation

In China’s official tourism resource evaluation criteria, the scoring and classification of individual tourism resource evaluation is explained in detail [6], but there is a contradiction of overlapping classes within the classification. Therefore, according to the clear hierarchy definition in China’s official tourism resource evaluation criteria, the health and wellness tourism resources are divided into five categories, and the expression discourse of the five types is improved. The value evaluation level of health and wellness tourism resources is divided into the following five categories.

1. The value score of health and wellness tourism resources is greater than or equal to 90 points, rated as fifth grade, and classified as super level.
2. The value score of health and wellness tourism resources is between 75 to 89 points, rated as fourth grade, and classified as excellent level.
3. The value score of health and wellness tourism resources is between 60 to 74 points, rated as third grade, and classified as good level.
4. The value score of health and wellness tourism resources is between 45 to 59 points, rated as second grade, and classified as ordinary level.
5. The value score of health and wellness tourism resources is between 30 to 44 points, rated as first grade, and classified as poor level.

## Discussion

### Reflections on China’s official tourism resource evaluation criteria

Between 1997 and 2017, China continuously explored and improved the evaluation standards of tourism resources, forming the latest official criteria of *Classification*, *Survey and Evaluation of Tourism Resources* (2017). Although some problems that appeared in previous tourism evaluation have been amended, China’s official tourism resource evaluation criteria is still characterized by the traditional understanding of tourism resources. First, the value evaluation index system is rough and blunt, which is not conducive to the in-depth value exploration of tourism resources. Second, in the evaluation of tourism resources, China’s official criteria adopts the direct scoring method of survey team to calculate evaluation level, which appears to be subjective and fails to take into account different opinions of stakeholders. Third, there is redundancy in its classification. For example, fifth-grade tourism resources are classified as special grade and excellent grade together with fourth grade and third grade ones.

Therefore, on the basis of critical reflection on China’s official tourism resource evaluation criteria, a value evaluation model of health and wellness tourism resources that is different from the official criteria is constructed, which is specifically reflected in the following three aspects. First, the characteristic elements of health and wellness tourism resources and public health incident plan are added in the evaluation system. Second, the opinions’ weight of administrators and tourists is included in score calculation. Third, the grade description discourse is improved.

In the evaluation process of health and wellness tourism resources, objective and subjective indicators should not be confused. The evaluation of objective indicators should be based on the real and objective analysis of ecological and environmental data and socio-economic data. As for the evaluation of subjective indicators, this study advocates for the design of a special questionnaire or interview outline to comprehensively consider the real experience of experts, administrators, and tourists for investigation, so that evaluation can be more objective.

### Reflections on the value evaluation of health and wellness tourism resources

Health and wellness tourism has been in dynamic development. With the evolution and upgrading of tourism and major public events, the value evaluation index will also change. Differing from existing studies, the index system constructed in this study adds the elements of health, wellness, and public health incident plan. The value evaluation research of health and wellness tourism resources should focus on long-term and dynamic analysis and make timely adjustments to the selection and modification of evaluation indicators based on national, social, and people’s conditions, so as to improve the scientific accuracy of the value evaluation on health and wellness tourism resources. In follow-up research, several different types of health and wellness tourism destinations can be selected for empirical and comparative study. It can also subdivide the types of tourists, further adjust the evaluation model through in-depth research, and enhance the universality and applicability of the evaluation system.

## Conclusions

Based on critical reflections of China’s official tourism resource evaluation criteria, this study establishes and improves the value evaluation system of health and wellness tourism resources from three aspects: construction of index system, selection of evaluation subjects, and grade discourse description.

In the aspect of index system construction, through two rounds of expert consultation, the health and wellness tourism resource evaluation system—including three first levels, seven second levels, and twenty-eight third levels—was finally established. The first layer covers three main value evaluation categories of health and wellness tourism resources. In the second layer, it not only absorbs standard elements of general tourism resources, but also adds the characteristic elements of health and wellness tourism resources. In the third layer, a number of indicators such as health value, culture, environment, location, and public health incident plans are taken into account. Stability of ecological environment, rehabilitation and convalescence value, and health preservation value are three key indicators to measure the values of health and wellness tourism resources. Tourism health value, environment quality, and construction conditions are the three key conditions to evaluate the values of health and wellness tourism resources.

In terms of evaluation subject selection, this study attaches importance to the different evaluation basis of multiple stakeholders and improves the Fishbein-Rosenberg evaluation model through the comprehensive perspective of experts, administrators, and tourists. It is applied to the value evaluation of health and wellness tourism resources to improve accuracy.

In terms of grade discourse description, the classification discourse of grade is modified into the following levels: super, excellent, good, ordinary, and poor. This description eliminates the redundancy of China’s official tourism resource evaluation criteria in the description of grade types, which is not only a rethinking of existing national criteria, but also a new attempt to research the value evaluation theory of health and wellness tourism resources.

In addition to the above contributions, this paper had some limitations in case application, because the evaluation index system has not been utilised in the field. Thus, future research should focus on the constant updating and application of the value evaluation index system of health and wellness tourism resources. Reasonable evaluation of health and wellness tourism resources can help correctly understand the characteristics of health and wellness tourism resources and promote related industries to carry out utilization work on this basis. The ultimate goal is to better meet the tourism needs of the elderly and sub-healthy people and respond to the national strategy. At the same time, it is the obligation of every scholar to reflect on the existing evaluation criteria used in China. Based on the inheritance and reflection, it is expected to promote the exchange of different views on tourism resources evaluation, stimulate the innovation of theories, and further improve the national criteria.
